# Colonic Mucosa-Associated Lymphoid Tissue Lymphoma Presented as Multiple Polyposis at Colonoscopy in a Nigerian Man: Case Report of a Rare Occurrence and Brief Review of Literature

**DOI:** 10.1200/JGO.2016.005124

**Published:** 2016-06-15

**Authors:** Aderemi Oluyemi, Nicholas Awolola

**Affiliations:** **Aderemi Oluyemi**, ReMay Consultancy and Medical Services, Ikeja; and **Nicolas Awolola**, University of Lagos, Idi-Araba, Lagos, Nigeria.

## INTRODUCTION

Anatomically, mucosa-associated lymphoid tissue (MALT) is found in various parts of the body. In the colon, lymphoid tissue occurs mainly as isolated lymphoid follicles that are composed mainly of B lymphocytes.^1,2^ Such gastrointestinal tract MALT plays an important role in immune surveillance and mucosal regeneration, but the cells in MALT may occasionally undergo abnormal proliferation and give rise to lymphoma of the MALT type.^1-3^ The World Health Organization identifies these lesions of non-Hodgkin lymphoma as being of B-cell origin and classifies them as extranodal marginal zone B-cell lymphoma of the MALT type.^4,5^

In 1983, Isaacson and Wright^6^ were the first to describe the term MALT lymphoma. In the gastrointestinal tract, the most common site of occurrence is the stomach and small intestine; only rarely does MALT occur in the colon.^7-9^ To put its rarity in context, all colonic lymphomas account for approximately 0.2% to 0.6% of colorectal malignant tumors and only 2.5% of all lymphomas.^7-11^ Among the lymphomas seen in the colon, the most common type is diffuse large B-cell lymphoma, and MALT-type lymphoma accounts for < 20% of cases in this subgroup.^12^

In the sub-Saharan region of Africa, the rarity of colonic MALT lymphoma is further highlighted by the dearth of published reports on the condition. Herein, we present one such rare case.

## CASE REPORT

**Fig 1:**
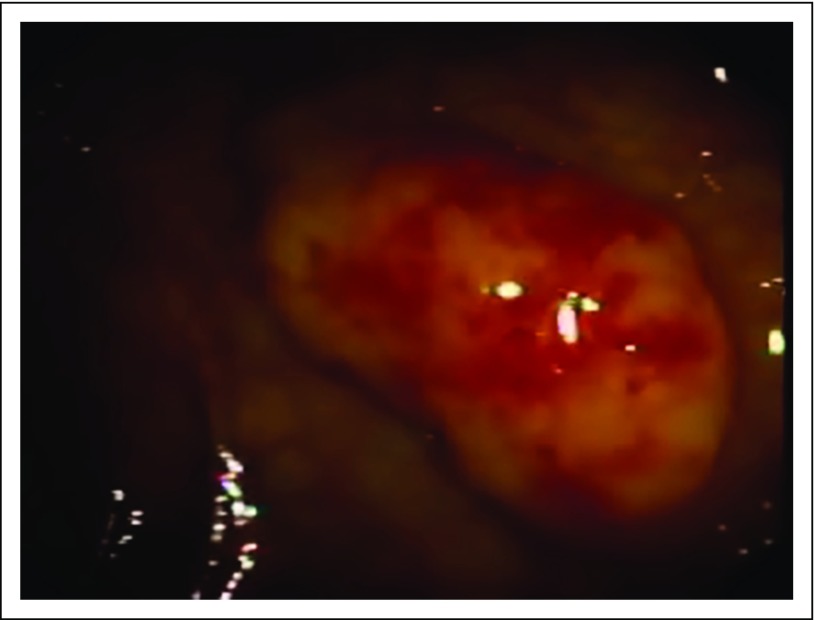
Image of rectal polyp at colonoscopy.

A 55-year-old Nigerian man was referred for a colonoscopy at a private center in Lagos, Nigeria, after a routine medical examination showed a positive fecal occult blood test. He had no family history of a gastrointestinal cancer and no history of hematochezia or dyschezia of any kind. He was asymptomatic at presentation. The patient’s earlier blood work included normal hemoglobin levels along with other normal parameters. His abdominal scan was also normal. The significant finding on colonoscopy was multiple polypoidal lesions of varying sizes scattered in isolation and in clusters across the rectum, sigmoid, and descending colon. The largest was an 8-mm diameter, semipedunculated lesion in the rectum (Fig 1). The smooth surface of this rectal lesion bore multiple dilated vascular structures.

**Fig 2:**
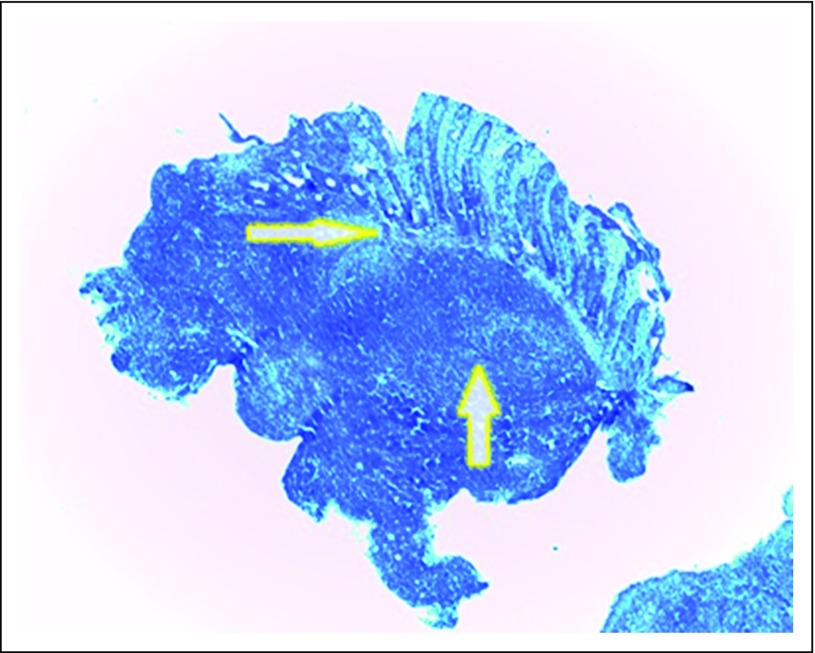
Low-power photomicrograph that shows sheets of dense and diffuse lymphocytic infiltrate with reactive germinal center (up arrow). This extended from the submucosa and invaded the muscularis and mucosa glands and destroyed the latter (lymphoepithelial lesion [right arrow]). The overlying mucosa was ulcerated (hematoxylin and eosin magnification, ×40).

**Fig 3:**
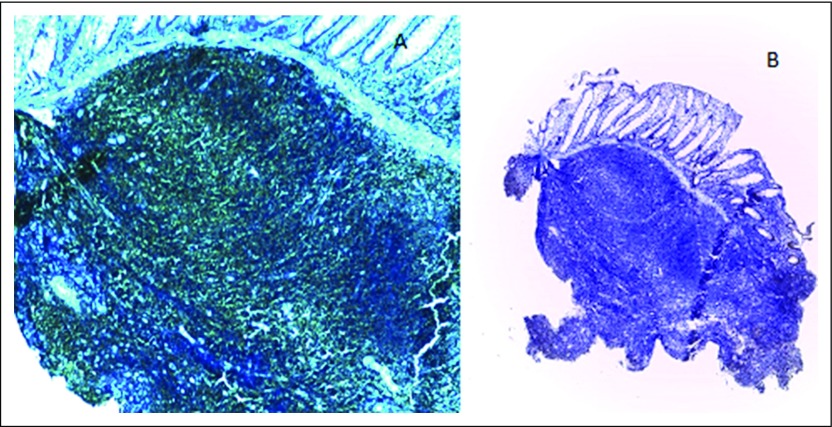
(A) Medium-power photomicrograph that shows a positive CD20 immunostain (×100). (B) Low-power photomicrograph that shows cyclin d1 negativity (×40).

**Fig 4:**
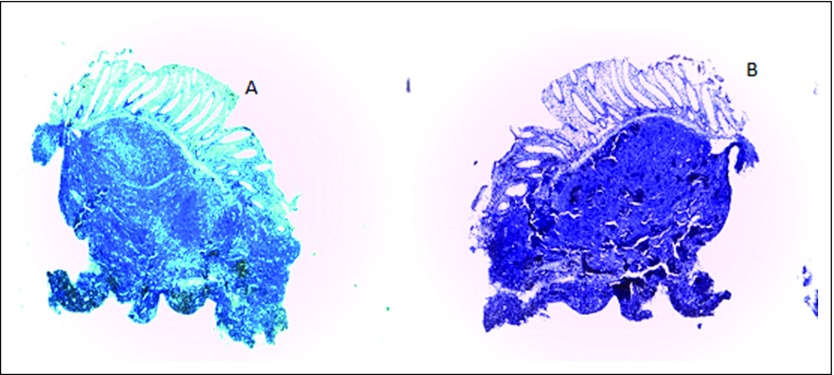
(A) Low-power photomicrograph that shows a negative CD3 immunostain (×40). (B) Low-power photomicrograph that shows CD5 negativity (×40).

**Fig 5:**
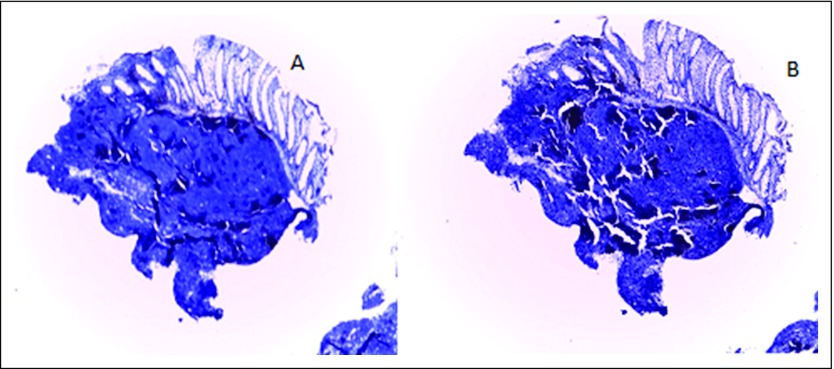
(A) Low-power photomicrograph that shows negative *bcl-2* (×40). (B) Low-power photomicrograph that shows CD10 negativity (×40).

Pathologic findings of the biopsy sample from the rectal lesion showed sheets of dense and diffuse lymphocytic infiltrate with a reactive germinal center. This extended from the submucosa and invaded the muscularis and mucosa glands and destroyed the latter (lymphoepithelial lesion). The overlying mucosa was ulcerated. The adjacent mucosa showed diffuse and moderate mixed inflammatory cells, including lymphocytes, plasma cells, and neutrophils in the lamina propria (Fig 2). No identifiable *Helicobacter*-like organisms were found. Further immunohistochemistry findings were markedly positive for CD20 and negative for CD3, CD5, CD10, cyclin D1, and *bcl-2* (Figs 3, 4, and 5).

A follow-up gastroscopy was normal. The only abnormality seen on a contrast-enhanced computed tomography scan of the entire abdominal and pelvic regions was localized symmetric thickening of the rectal wall with no evidence of local infiltration or lymphadenopathy at any other site. Thus, the diagnosis of colonic MALT lymphoma was made. The patient was advised to have a specialist surgical and radiotherapy oncologic review with a view to definitive management of his condition, but the referring physician reported that the patient declined and has not been heard from since.

## DISCUSSION

Primary colorectal lymphoma comprises low-grade B-cell lymphoma that arises from diffuse large B-cell, MALT, mantle cell, and T-cell lymphomas.^13^ These are rare tumors that constitute only 0.2% of all malignant tumors that arise from the colorectal region, with the MALT type accounting for < 20% of cases.^12,14^ Thus, the novelty of this case report underlines that such occurrence from the sub-Saharan region of Africa is particularly rare.

Clinical presentation ranges from asymptomatic and discovered only through routine tests (as was the case in the current patient) to presentation with nonspecific abdominal complaints to more dramatic presentations that include profuse bleeding from the rectum, intestinal obstruction, and intussusceptions.^1,15^ The endoscopic appearance of these lesions also varies widely from protruding or ulcerative masses that appear either singly or in clusters to multiple widespread masses with diffuse pancolonic involvement.^10,16^ The current case is an example of another type of multiple polypoidal appearance that is restricted to the colon.^17^

A critical morphologic characteristic of MALT lymphomas is the simulation of normal MALT.^18,19^ The neoplastic B lymphocytes of MALT lymphomas are found in marginal zones that adjoin reactive follicles and often in diminished rims of mantle zone lymphocytes. The marginal zone cells of MALT invade not only residual reactive follicles but also the epithelium. Epithelial invasion with frequent destruction by the B cells of MALT has been referred to as a lymphoepithelial lesion and is a vital morphologic attribute in the diagnosis of many MALT-derived lymphomas.^18,19^ These features were clearly demonstrated in the current patient and prompted further elucidation with immunohistochemistry.

The marginal zone cells of MALT share immunophenotypic characteristics. MALT lymphomas are B-cell derived with CD20 expression and frequently contain numerous admixed CD3^+^-reactive T cells.^18,19^ Unlike chronic lymphocytic leukemia/small lymphocytic lymphoma and mantle cell lymphoma, the lymphomas of MALT origin usually lack CD5 and are without *bcl-2* gene rearrangements.^20^ MALT lymphomas differ from follicular lymphomas in that they are negative for CD10 and do not exhibit *bcl-2* rearrangements.^20^ The immunophenotypic results of the current biopsy samples along with the characteristic histologic features led to the confirmation of MALT lymphoma.

Treatment of lymphoma tends to be gratifying because unlike their nodal cousins, lymphomas tend to be localized at the time of diagnosis and may be effectively treated with local therapy. However, because of the lack of an accepted etiology and given their rarity, little consensus exists on the optimal treatment regimen of colonic MALT lymphomas.^15,21^ Antibiotic treatment of *Helicobacter pylori* is controversial because studies exist in support of and against its usefulness.^22-24^ Various chemotherapeutic agents have been tried with some degree of response, but there remains no standardized therapy.^15^ Surgical resection may be effective when a colorectal MALT lymphoma does not respond to *H. pylori* eradication therapy or chemotherapy and is localized without dissemination.^15,24^

Important insight to gain from this report is that the absence of structured colorectal screening programs in sub-Saharan Africa has cost us the ability to detect more of such findings in the asymptomatic population. Thus, this has denied patients the benefit of early detection and intervention in more amenable neoplastic lesions.
